# Diverse anti-defence systems are encoded in the leading region of plasmids

**DOI:** 10.1038/s41586-024-07994-w

**Published:** 2024-10-09

**Authors:** Bruria Samuel, Karin Mittelman, Shirly Ynbal Croitoru, Maya Ben Haim, David Burstein

**Affiliations:** The Shmunis School of Biomedicine and Cancer Research, George S. Wise Faculty of Life Sciences, Tel-Aviv University, Tel-Aviv, Israel

**Keywords:** Sequence annotation, Data processing, Metagenomics, Bacterial genomics, Bacterial genetics

## Abstract

Plasmids are major drivers of gene mobilization by means of horizontal gene transfer and play a key role in spreading antimicrobial resistance among pathogens^1,2^. Despite various bacterial defence mechanisms such as CRISPR–Cas, restriction–modification systems and SOS-response genes that prevent the invasion of mobile genetic elements^3^, plasmids robustly transfer within bacterial populations through conjugation^4,5^. Here we show that the leading region of plasmids, the first to enter recipient cells, is a hotspot for an extensive repertoire of anti-defence systems, encoding anti-CRISPR, anti-restriction, anti-SOS and other counter-defence proteins. We further identified in the leading region a prevalence of promoters known to allow expression from single-stranded DNA^6^, potentially facilitating rapid protection against bacterial immunity during the early stages of plasmid establishment. We demonstrated experimentally the importance of anti-defence gene localization in the leading region for efficient conjugation. These results indicate that focusing on the leading region of plasmids could lead to the discovery of diverse anti-defence genes. Combined, our findings show a new facet of plasmid dissemination and provide theoretical foundations for developing efficient conjugative delivery systems for natural microbial communities.

## Abstract (web summary)

The leading region of plasmids is a hotspot for many anti-defence systems, encoding anti-CRISPR, anti-restriction and other counter-defence proteins; focusing on this region could lead to the discovery of diverse anti-defence genes, and improve strategies for the design of plasmid-based delivery systems for diverse microbial ecosystems.

## Main

Conjugation is a primary horizontal gene transfer mechanism in which DNA is transferred between microbial cells. Conjugative plasmids drive rapid bacterial evolution^7^ and present a major challenge in combating the spread of antimicrobial resistance genes (ARGs)^1,2^.

Conjugative elements’ transport machinery comprises type IV secretion system (T4SS) proteins, an origin of transfer (*oriT*) and a relaxosome (a relaxase, often with auxiliary proteins)^8^. Whereas conjugative plasmids and integrative conjugative elements (ICEs) encode for the entire transport machinery, mobilizable plasmids, containing only relaxosome components and *oriT* (refs. ^9,10^), rely on coresiding conjugative elements for transfer^11^. We refer to both types as ‘potential conjugative elements’, as they can be transferred by conjugation^12^. Conjugation is initiated with the assembly of the relaxosome at the *oriT* and the nicking of the *nic* site within the *oriT* (ref. ^8^). The nicked DNA strand (T-strand) is transferred into the recipient cell, and the first transferred region is termed the leading region. The relaxase is typically located in the lagging region, which enters the recipient cell last^13,14^.

Previous studies suggested that the leading region genes are important for plasmid establishment during conjugation^15,16^. In certain plasmids, these genes are expressed early on entry into the recipient cell^17–19^, preceding the conversion of the entering single-stranded DNA (ssDNA) into double-stranded DNA (dsDNA)^20^. The regulation of some of these genes involves unique promoters designated F*rpo*, which adopt a secondary structure that mimics a double-strand conformation, allowing recognition by the host RNA polymerase^6,17^. Thus, F*rpo* functions as a single-strand promoter, enabling early expression of leading region genes^20^.

Conjugative elements face various prokaryotic defence systems, including restriction–modification and CRISPR–Cas^3,21^. Despite these defences designed to prevent the entry of exogenous DNA, horizontal gene transfer widely persists across bacterial species^4,5^. This is enabled, among other factors, by anti-defence mechanisms such as anti-restriction and anti-CRISPR genes developed by mobile genetic elements (MGEs)^22,23^. A few of the genes described in plasmids’ leading regions encode anti-defence proteins, such as ArdA, an anti-restriction protein^24^, and PsiB, which inhibits the bacterial SOS response^25^, and are known to be early expressed^18^. However, these studies were performed on very few genes and plasmids (IncI, ColIb-P9 and F plasmids). Most of the leading region genes and their function during conjugation remain largely unexplored^26,27^.

We investigated the leading region’s role in conjugative elements’ ability to evade host defences. We proposed that for anti-defence genes to be effective, they need to be rapidly expressed in the very early stages of conjugation^28^, reminiscent of early expression of anti-CRISPRs and the *ocr* anti-restriction gene reported in phages^29,30^. In line with this hypothesis, we discovered that the leading regions of conjugative elements are highly enriched with anti-defence genes and that these regions contain various uncharacterized genes, many of which are probably anti-defence related. Our results indicate that the leading regions act as ‘anti-defence islands’, protecting conjugative elements from host defences upon entry to recipient cells.

## Anti-defence genes in the leading region

**Extended Data Fig. 1:**
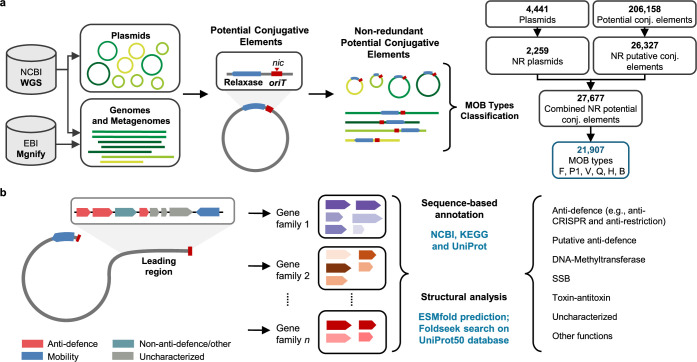
Workflow overview. **a**, All assembled genomes and metagenomes available in NCBI’s^61^ and EBI’s^62^ databases were analysed. In the first phase, we considered only sequences explicitly annotated as plasmids. In the second phase, we included all sequences that contained a detectable relaxosome component gene in proximity to a known *oriT* sequence. We termed the combined set of the two phases “potential conjugative elements”. Redundant elements of this set were omitted based on sequence similarity. The non-redundant sequences were then classified according to their MOB types. **b**, We mapped to the potential conjugative elements the leading and lagging regions, known anti-defence genes (anti-restriction, anti-CRISPR, and anti-SOS), and transfer-related genes. We focused on the genes enriched in the leading region and characterized them further. These gene families were classified based on sequence and structural similarity, into the following groups: anti-defence, putative anti-defence, DNA-methyltransferases, SSB genes, toxin-antitoxin genes, uncharacterized genes, and other functional families.

We analysed all sequences annotated as plasmids in the National Center for Biotechnology Information’s (NCBI) Whole-Genome Shotgun (WGS) database to explore the position of anti-defence genes relative to the *oriT* (workflow overview in Extended Data Fig. 1a). We focused on plasmids with an *oriT* adjacent to a relaxase/*traM* gene, allowing us to discern the leading and lagging regions, as these relaxosome genes are typically encoded in the lagging region near the *oriT* (refs. ^13,14^) (Extended Data Fig. 1b). The plasmids were detected by seeking homology to experimentally validated and predicted *oriT*s, followed by locating a relaxase/*traM* relaxosome gene and anti-defence genes using profile hidden Markov models (pHMMs). Anti-defence profiles included anti-CRISPRs antagonizing CRISPR–Cas systems^22^, anti-restriction proteins inhibiting restriction–modification endonucleases^23,31^ and SOS inhibitors suppressing the host SOS response elicited by plasmid entry.

**Extended Data Fig. 2:**
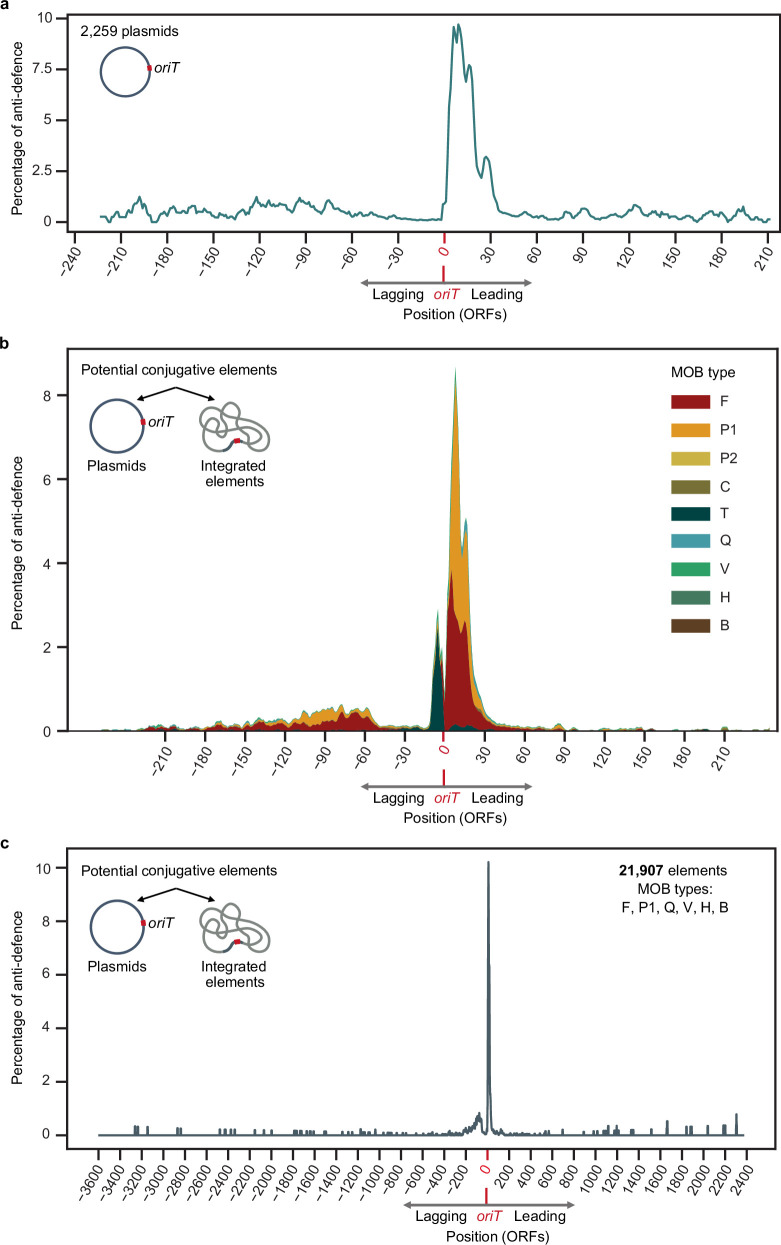
Frequency of anti-defence genes relative to the origin of transfer (*oriT*). **a**, Anti-defence gene frequency in 2,259 non-redundant sequences annotated as plasmids. The *x*-axis shows ORF indices relative to the *oriT*, with 0 representing the first ORF in the leading region. Positions that were represented in at least 150 sequences annotated as plasmids are plotted. The *y*-axis indicates the average frequency of anti-defence genes (combining SOS inhibition, anti-restriction, and anti-CRISPR genes) over a five ORF window. **b**, Analysis of 22,897 out of 26,327 non-redundant potential conjugative elements that could be reliably mapped to a MOB type. The *x*-axis shows ORF indices relative to the *oriT*, with 0 representing the first ORF in the leading region. The *y*-axis indicates the average frequency of anti-defence genes (combining SOS inhibition, anti-restriction, and anti-CRISPR genes) over a five-ORF window, with frequencies for each MOB type colour-coded and stacked. Only positions represented in at least 1,000 sequences are shown. **c**, Anti-defence gene frequency within the 21,907 potential conjugative elements retrieved from genomic and metagenomic databases (MOB types F, P1, Q, V, H, B). Only positions represented in at least 50 sequences are shown. Source Data

Measuring the relative abundance of anti-defence genes at each position relative to the *oriT* location revealed that leading regions are highly enriched with anti-defence genes (Extended Data Fig. 2a). Specifically, most of the first 30 open reading frames (ORFs) in the leading regions were significantly enriched with anti-defence genes (one-sided Fisher’s exact test, *α* = 0.001).

To assess the generality of this phenomenon, we expanded the dataset beyond explicitly annotated plasmids (which did not include ICEs and were biased towards pathogens and model organisms). We thus searched all publicly available genomic and metagenomic assemblies from NCBI and European Bioinformatics Institute (EBI) for potential conjugative elements by identifying contigs with relaxase/*traM* genes in proximity to an *oriT*. Within the 26,327 additional non-redundant potential conjugative elements detected, we again observed anti-defence gene enrichment in the leading region. However, a notable proportion of these genes was also identified in the lagging region (Extended Data Fig. 2b). Dissecting the dataset by mobilization (MOB) types showed significant enrichment (one-sided Fisher’s exact test, *α* = 0.001) of anti-defence genes in leading regions across most MOB types. However, MOB_T_, MOB_P2_ and MOB_C_ showed no discernible anti-defence gene enrichment in the leading regions (Extended Data Fig. 2b) and were omitted from downstream analysis. Notably, uneven distribution of MOB types across bacterial phyla and mobile element types^32^ suggests variations in conjugation mechanisms and interactions between host defences and plasmid anti-defences. For instance, the MOB_T_ type, common in ICEs and widely distributed within Firmicutes^33^, shows unique relaxase characteristics^34^ that may influence the function of the leading region.

**Fig. 1:**
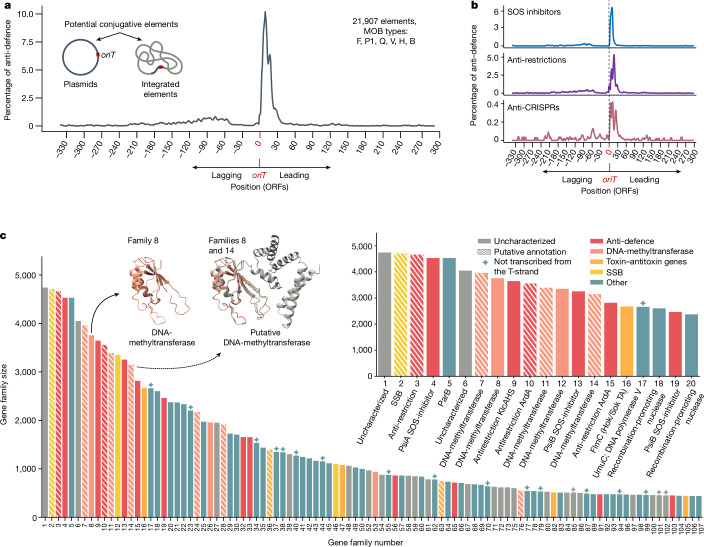
Enrichment of anti-defence genes in the leading region. **a**, Anti-defence gene frequency in 21,907 plasmids and potential conjugative elements (MOBs F, P1, Q, V, H, B). The *x* axis shows ORF indices relative to the *oriT*, with 0 representing the first ORF in the leading region. Only positions represented in at least 500 sequences are shown (additional positions in Extended Data Fig. 2c). The *y* axis indicates the average frequency of anti-defence genes (combining SOS inhibition, anti-restriction and anti-CRISPR genes) over a five-ORF window. **b**, Breakdown of anti-defence gene frequency by functional category. **c**, The 100 largest gene families significantly enriched in the leading regions (one-sided Fisher’s exact test, *α* = 0.001 after FDR correction for multiple testing), categorized into six groups: (1) anti-defence genes, which are anti-CRISPRs, anti-restriction genes and SOS inhibitors; (2) DNA methyltransferases (MTases); (3) toxin–antitoxin genes; (4) SSBs; (5) other, which are annotated genes with no known association to anti-defence and (6) uncharacterized genes. The *y* axis shows the family size, whereas the *x* axis shows the families ranked on the basis of their size. Note that some of the most prevalent families are not enriched specifically in the leading region and are thus omitted from this analysis. Diamonds indicate gene families encoded opposite to the T-strand, which cannot be transcribed from the leading ssDNA. Putative annotations are indicated with striped bars. Structural comparison between a DNA-methyltransferase from *Escherichia coli* (gene family 8, NCBI accession CP029982.1) and a putative DNA-methyltransferase from *E. coli* (gene family 14, NCBI accession MCJK01000027.1) are presented above the respective families. The inset focuses on the 20 largest gene families (Supplementary Table 1). Source Data

**Extended Data Fig. 3:**
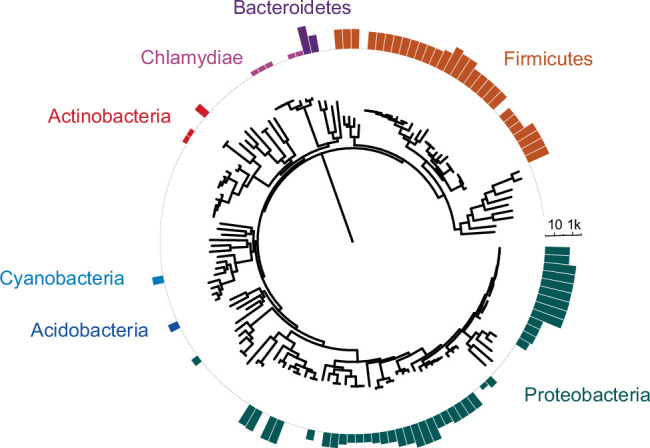
Phylogenetic distribution of the analysed conjugative elements. The phylogenetic distribution of the 13,738 non-redundant plasmids and potential conjugative elements. This set excluded 8,169 elements originating from metagenomes and sequences that could not be reliably mapped to the tree. The bacterial tree of life was acquired from iTOL^75^, with bars colour-coded according to phyla, representing the conjugative element count on a log_10_ scale.

The combined set of well-characterized plasmids and potential conjugative elements comprised 27,677 non-redundant sequences. Excluding the three MOB types with no significant enrichment in the leading region left 21,907 sequences. In this dataset, most of the 29 leading positions were significantly enriched with anti-defence genes (Fig. 1a and Extended Data Fig. 2c; one-sided Fisher’s exact test, *α* = 0.001). Analyses of each anti-defence category showed similar trends (Fig. 1b). This demonstrates that, across diverse conjugative elements from a wide range of bacterial hosts (Extended Data Fig. 3), the genes first transferred are disproportionately enriched with anti-defence functions.

## Roles of abundant leading region genes

To better understand the function of prevalent gene families in the leading regions, we clustered genes from anti-defence-enriched locations within the 21,907 non-redundant conjugative elements. We then identified gene families specifically enriched in the leading region, by comparing their prevalence to other regions on the same contigs. This analysis revealed that 255 of the 300 largest families were significantly enriched in the leading region (one-sided Fisher’s exact test, *α* = 0.001, Supplementary Table 1).

Focusing on the 100 largest gene families significantly enriched in the leading region revealed three main functional groups beyond known anti-defence genes (Fig. 1c). One of the most prominent functions was ‘orphan’ DNA-methyltransferases (MTases), potentially protecting conjugative elements from host restriction–modification systems. This protective role of orphan MTases has been previously demonstrated in phages^35–37^ and more recently also observed in plasmids in which MTases encoded on the pESBL plasmid methylate entering ssDNA early in conjugation^38^.

SSBs (ssDNA-binding proteins) were also frequently encoded in these regions, often adjacent to SOS inhibitors (*psiA* and *psiB*). Plasmid-encoded SSBs are important for effective SOS inhibition by PsiB^39,40^ and may aid in evading host CRISPR–Cas systems by facilitating dsDNA break repair^41^. SSBs also protect ssDNA intermediates from nuclease degradation and interact with various bacterial genome maintenance proteins, including recombination, repair and replication factors^42^. These functions suggest multiple protective roles during early conjugation stages, alongside other possible roles in the newly transconjugant cells, including involvement in plasmid duplication^20^.

Toxin and antitoxin genes, both as part of complete toxin–antitoxin systems and as orphan antitoxins, were also highly represented in the leading regions. Although toxin–antitoxin systems are encoded throughout conjugative element genomes, their overrepresentation in leading regions suggests a potential protective role in conjugative element establishment (Supplementary Discussion).

**Extended Data Fig. 4:**
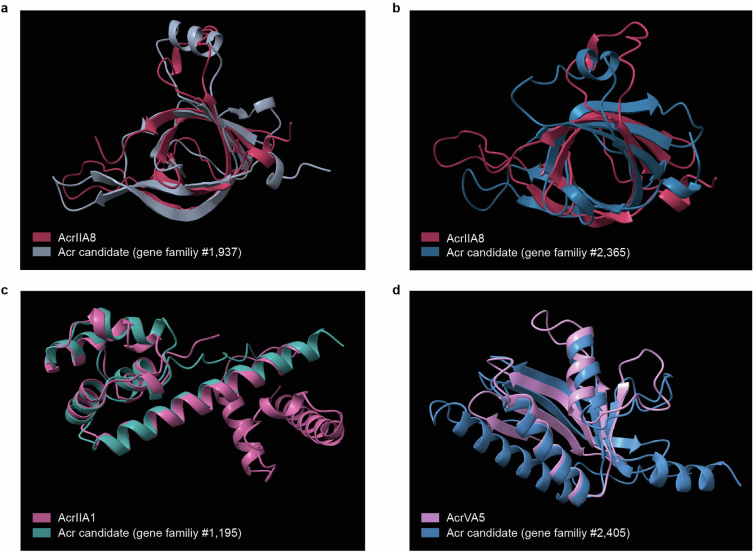
Structural comparison of known anti-CRISPRs and anti-CRISPR candidates identified based on their location and structural similarity. **a**, AcrIIA8 anti-CRISPR (NCBI accession VDB32352.1) compared to a putative anti-CRISPR found in a conjugative element from a human gut metagenome (Mgnify analysis accession ERZ1741958, NODE_63). **b**, Anti-CRISPR AcrIIA8 (NCBI Protein accession VDB32352.1) compared to a putative anti-CRISPR found in a conjugative element of *Staphylococcus epidermidis* (NCBI accession VYVG01000002.1). **c**, Anti-CRISPR AcrIIA1 (NCBI accession WP_003722518.1) compared to a putative anti-CRISPR found in a conjugative element from a human gut metagenome (NCBI accession BABC01000244.1). **d**, Anti-CRISPR AcrVA5 (NCBI accession WP_046699157.1) compared to a putative anti-CRISPR found in a conjugative element of *Salmonella enterica* (NCBI accession AAEVVI010000002.1).

Notably, 33% of the 100 most prevalent gene families in leading regions were uncharacterized (Fig. 1c). Given the considerable overrepresentation of anti-defence genes in this region, many of these families probably have anti-defence-related functions. Investigating the largest unannotated families revealed potential anti-defence roles (Extended Data Table 1 and Supplementary Table 1). To further explain these functions, we conducted structural analyses of 107,893 proteins belonging to uncharacterized families enriched in the leading region and primarily encoded on the T-strand. This analysis uncovered potential anti-defence genes undetectable by sequence similarity, including putative anti-CRISPRs (for example, *acrIIA8*, *acrVA5* and *acrIB*) and anti-restriction genes (for example, *darA* and *ardA*, Extended Data Fig. 4 and Extended Data Table 1). The structural analysis further underscored the prevalence of MTases, SSBs and toxin–antitoxin genes within the leading region of plasmids. Phage-associated annotations were found in nearly 10% of the analysed gene families, suggesting shared anti-defence mechanisms between plasmids and phages.

## Anti-defence islands

**Fig. 2:**
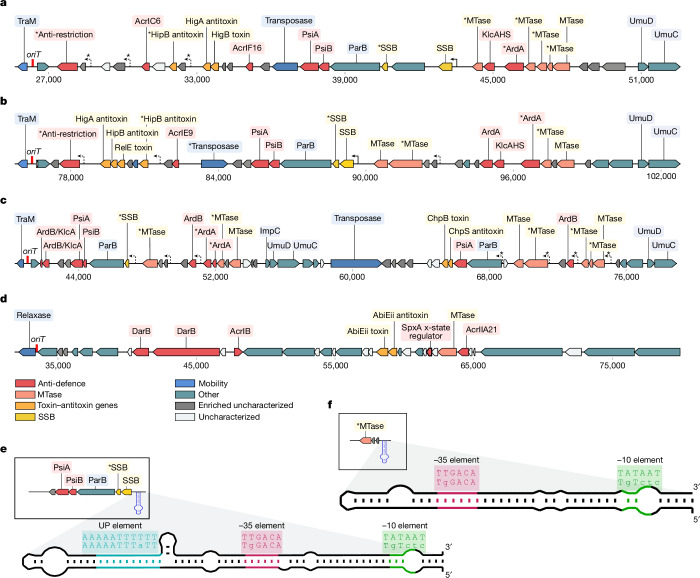
Representative anti-defence islands and ssDNA promoters. **a**–**d**, Representative anti-defence islands from leading regions of conjugative elements in: (**a**) *Salmonella enterica* (NCBI assembly accession AAEPNF010000010.1), (**b**) *Serratia* marcescens (NCBI accession CP047692.1), (**c**) an insect metagenome (NCBI accession OFEI01000013.1) and (**d**) *Streptococcus pneumoniae* (NCBI accession CPMX01000004.1). The *oriT* location is marked in red on the left. Genes are colour-coded by functional category: anti-defence (red), MTase (peach), toxin–antitoxin genes (orange), SSB (yellow), mobility (transfer genes, blue), other (gene without known association to anti-defence, teal), uncharacterized genes enriched in the leading regions (grey), other uncharacterized genes (white). Asterisks (*) next to gene annotations indicate a potential anti-defence function. F*rpo* promoters are indicated by arrows: a solid arrow for promoters with significant similarity to known F*rpo* sequences, a dashed arrow for F*rpo* candidates and dashed with an asterisk (*) for low-certainty candidates. Further islands are presented in Extended Data Fig. 5. **e**, Predicted secondary structure of the F*rpo* in *S. marcescens* plasmid from **b**. **f**, Putative F*rpo* candidate in the conjugative element from **c**. Regions corresponding to the −10, −35 and UP elements, as well as their complementary regions, are coloured and highlighted above the structure, along with the canonical sequences of these elements. Uppercase letters indicate nucleotides conforming to canonical sequences of the −35 and −10 elements (5′-TTGACA-3′ and 5′-TATAAT-3′, respectively; full sequences in Extended Data Fig. 6b,c).

**Extended Data Fig. 5:**
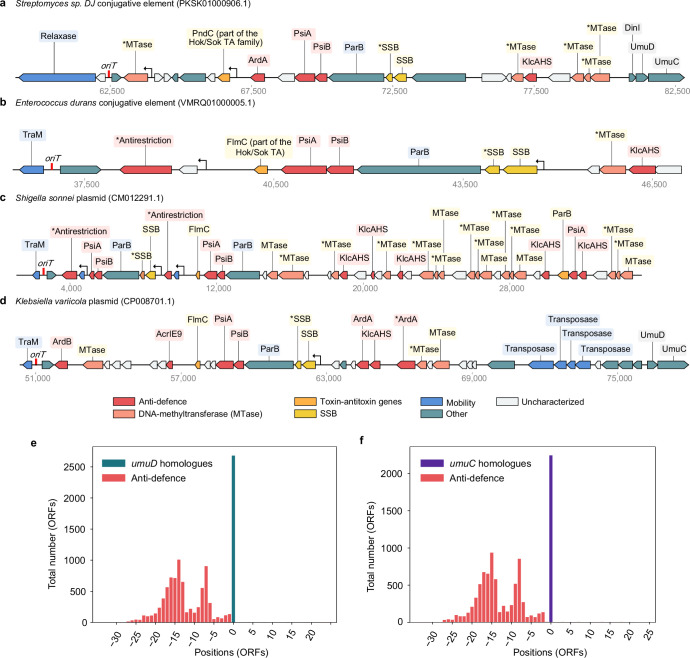
Additional examples of anti-defence islands from various bacterial hosts. **a**–**d**, Islands from leading regions of conjugative elements in: (**a**) *Streptomyces sp. DJ* (NCBI accession PKSK01000906.1), (**b**) *Enterococcus durans* (NCBI accession VMRQ01000005.1), (**c**) *Shigella sonnei* (NCBI accession CM012291.1) and (**d**) *Klebsiella variicola* (NCBI accession CP008701.1). The *oriT* location is marked in red on the left. Genes are coloured-coded by functional category: anti-defence (red), DNA-methyltransferase (MTase, peach), toxin-antitoxin genes (orange), ssDNA-binding protein (SSB, yellow), mobility (transfer genes, blue), other (gene without known association to anti-defence, teal). F*rpo* promoters are indicated by an arrow. Asterisks (*) indicate unannotated genes with a putative anti-defence function. **e**, Position distribution of anti-defence genes at each ORF position relative to *umuD* homologues (set as position 0) in the leading region. **f**, Similar analysis for *umuC* homologues. In cases of multiple *umuD*/*umuC* genes in the same leading region, the homologue closest to the *oriT* was used as reference.

We noticed that anti-defence genes in plasmids’ leading region tended to cluster into islands (Fig. 2a–d and Extended Data Fig. 5a–d), as previously reported for MGEs with clustered anti-defence genes^43^. We refer to these as islands because most annotated genes in these clusters share similar functions and reside between defined boundaries: the *oriT* on one end and often *umuCD* homologues on the other (Extended Data Fig. 5e,f). These islands contained different combinations of adjacent anti-defence genes and genes potentially protecting invading DNA such as MTases and SSBs. For example, we identified an island in the leading region of a *Salmonella enterica* conjugative element containing two anti-CRISPRs (*acrIC6* and *acrIF16*) near an anti-restriction (*klcAHS*), SOS inhibitors (*psiA* and *psiB*), MTases, SSBs and a toxin–antitoxin system (*higB-higA*, Fig. 2a). A similar island in the leading region of a *Serratia marcescens* plasmid harboured an anti-CRISPR inhibiting a different type of CRISPR–Cas system (*acrIE9*) and an additional antitoxin gene (*hipB*, Fig. 2b). The *hipB* antitoxin, typically countering HipA toxicity as part of *hipBA* operons^44^, was found next to a *higA*/*relE* toxin–antitoxin system in this island. It may function as an orphan antitoxin inhibiting competitive MGEs or host toxin–antitoxin defence systems (Supplementary Discussion).

Many of the islands were flanked by an operon of *umu*-like genes, forming the island’s terminating boundary (Extended Data Fig. 5e,f). These genes are plasmid homologues of *umuC* and *umuD*, which encode chromosomal translesion DNA synthesis polymerases (DNA polymerase V)^45^. Although widespread in conjugative elements^46,47^ and other MGEs, including the conjugative transposon Tn5252 (ref. ^48^) and phages^49,50^, their role in plasmids remains unclear^51^. Despite their high abundance in the leading region, 90.3% are not oriented for transcription from the T-strand, suggesting they are not expressed early in conjugation. Notably, one of the anti-defence islands we detected seemed to consist of two adjacent islands separated by a transposase, with *umu*-like gene operons flanking each of these adjacent islands (Fig. 2c).

Using uncharacterized gene families enriched in the leading regions, we detected more putative anti-defence islands. One such island, originating from a conjugative element from the Gram-positive pathogen *Streptococcus pneumoniae*, included two anti-CRISPRs (*acrIB1* and *acrIIA21*), two *darB* anti-restriction genes, an MTase, a toxin–antitoxin system (*abiEii*-*abiEi*), two uncharacterized gene families prevalent in leading regions and an *spxA* gene (Fig. 2d). SpxA represses X-state, a stress-response mechanism inducing competence in *S. pneumoniae* (a species lacking a classical SOS-response pathway)^52^. MGEs reportedly disrupt competence genes^53,54^, preventing exogenous DNA uptake that could presumably contribute to MGE elimination^55^. The plasmid-encoded SpxA may thus serve as an ‘anti-X-state’ protein preventing stress response, akin to SOS inhibitors found in other plasmids.

## ssDNA promoters in anti-defence islands

Analysis of the 300 most prevalent gene families enriched in the leading region showed that all anti-defence genes, MTases, SSBs and toxin–antitoxin genes were encoded exclusively on the T-strand (Fig. 1c). This orientation suggests potential transcription from the strand first transferred to the recipient, even before synthesis of the plasmid’s complementary strand.

Specific promoters, known as F*rpo* or *ssi*, which create secondary DNA structures mimicking dsDNA, can facilitate transcription from ssDNA^17,38^. We searched known F*rpo*/*ssi* sequences in the leading regions of the 21,907 potential conjugative elements, detecting 13,089 F*rpo*-homologous promoters in 6,006 conjugative elements. In the leading regions of *S. enterica* and *S. marcescens* plasmids, we identified one F*rpo-*like sequence immediately upstream of an SSB gene (Fig. 2e). Notably, F*rpo* transcription is highly stimulated by SSB^6^. These F*rpo* sequences in *S. enterica* and *S. marcescens* show roughly 89 and 80% identity, respectively, with an F plasmid F*rpo* upstream of an SSB gene demonstrated to be early transcribed from ssDNA^20^.

**Extended Data Fig. 6:**
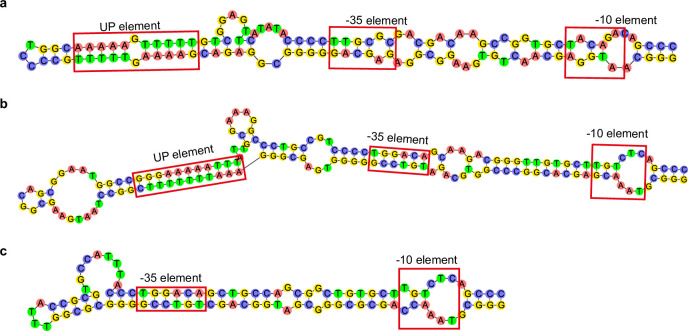
Sequences and predicted secondary structures of F*rpo* promoters. **a**, Candidate Frpo* sequences found in *S. enterica* conjugative elements (Fig. 2a), showing limited similarity to known F*rpo*. Promoter elements (−10, −35, and UP) are indicated by red boxes. **b**, F*rpo* identified upstream of an SSB protein in *S. marcescens* plasmid (see Fig. 2b). **c**, Candidate F*rpo*’ detected in a conjugative element from an insect gut metagenome (Fig. 2c). Sequences in **b**,**c** exhibit high sequence similarity to known F*rpo* sequences.

In the insect metagenome and *S. pneumoniae* islands, no sequences with significant similarity to F*rpo* were found. We thus conducted a more sensitive search for F*rpo*-like candidates upstream of ORFs in these islands, on the basis of the conformance of the predicted secondary structure with known F*rpo*s and the consensus sequences of the −35 and −10 elements (Supplementary Table 2). In the *S. marcescens* island, we detected three F*rpo-*like candidates (F*rpo*’). The search in the *S. enterica* island yielded three sequences bearing only distant F*rpo* similarity (Frpo*), showing secondary structures similar to known F*rpo* but considerable differences in the conserved −35 and −10 elements (Extended Data Fig. 6a). Analysis of the insect metagenome island led to the detection of three F*rpo*-like candidates (F*rpo*’, Fig. 2f) and four putative F*rpo* candidates with only distant similarity to known F*rpo* sequences (Frpo*). We next searched for the F*rpo*-like candidates (F*rpo*’ and Frpo*) within the entire set of leading regions. This analysis identified 7,751 F*rpo*’ and 950 Frpo* candidates, presenting high and limited similarity to F*rpo* sequences, respectively. Overall, examination of regions upstream of ORFs in the islands revealed a widespread presence of F*rpo*-like promoters in anti-defence islands, suggesting they potentially allow early expression from ssDNA during the initial stages of conjugation.

## Impact of leading genes on conjugation

**Fig. 3:**
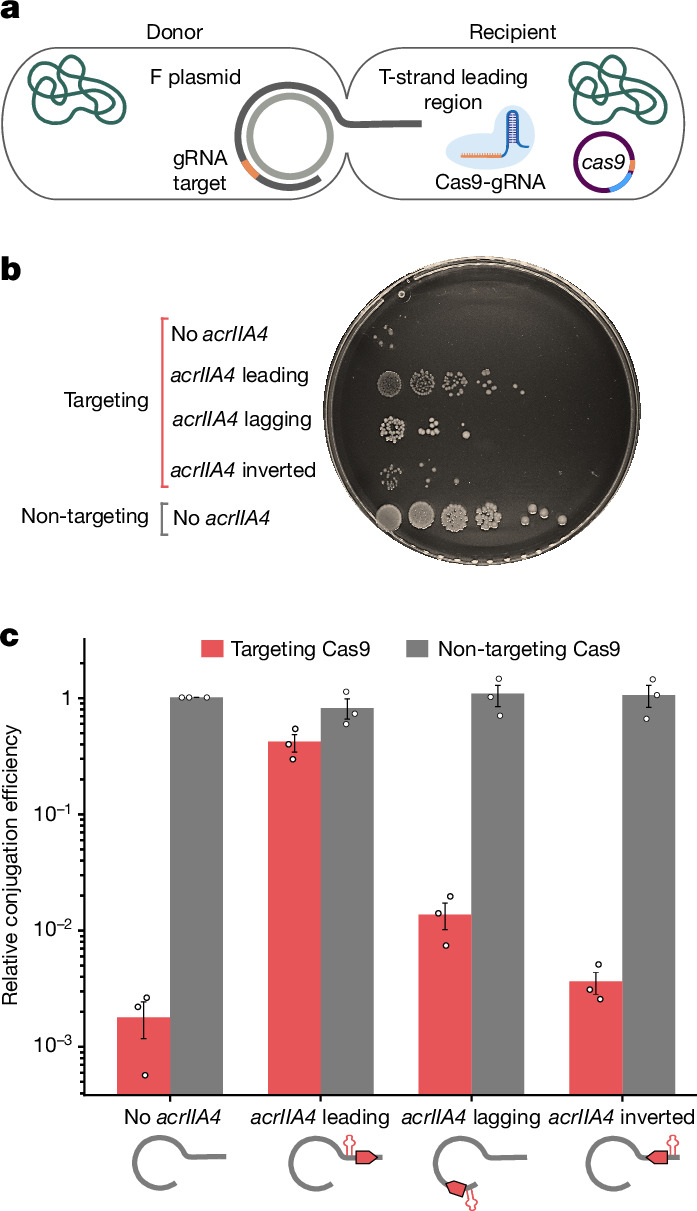
The effect of anti-CRISPR in the leading region on conjugation efficiency. **a**, Schematic representation of the donor and recipient cells during the conjugation experiments. The F plasmid’s T-strand is transferred into the recipient, starting with its leading region. In the recipient, a separate plasmid expresses Cas9 targeting the F plasmid. **b**, Representative example of transconjugant cell growth for each F plasmid variant. Droplet rows represent serial 1:5 dilutions. **c**, Conjugation efficiency as a function of the positioning and orientation of the anti-CRISPR *acrIIA4* on the F plasmids. Bars indicate the mean conjugation efficiency of each F plasmid variant relative to the control, which is an F plasmid with no anti-CRISPR gene transferred to a recipient with a non-targeting gRNA. Red bars represent recipients with a targeting gRNA, whereas grey bars represent recipients with a non-targeting gRNA. Conjugation efficiency is calculated as the transconjugant frequency (T/(R + T)) per conjugation, divided by the transconjugant frequency of the control (T, transconjugants and R, recipient cells). Data are presented as mean values ± s.e.m. from *n* = 3 biologically independent experiments. Individual data points from each experiment are overlaid on the corresponding bars. Source Data

We experimentally investigated how positioning anti-defence genes in the leading region of conjugating plasmids’ T-strand affects conjugation efficiency when the recipient bacteria contain a defence system (Fig. 3a). Specifically, we tested conjugation efficiencies of four F plasmid variants transferred to recipients expressing Cas9: (1) with an anti-CRISPR (*acrIIA4*) under an F*rpo* promoter in the T-strand’s leading region; (2) with an anti-CRISPR and an F*rpo* in the T-strand’s lagging region; (3) with an anti-CRISPR and an F*rpo* in the leading region of the T-strand’s complement and (4) with no anti-CRISPR. We used two recipients: one with a guide RNA (gRNA) targeting the F plasmid, and another with a non-targeting gRNA as a negative control.

In the absence of the anti-CRISPR, Cas9 strongly inhibited conjugation in a guide-dependent manner: non-targeted plasmids transferred roughly 550 times more efficiently than targeted plasmids. Plasmids encoding the anti-CRISPR in the leading region under an F*rpo* promoter effectively overcame Cas9 inhibition, resulting in conjugation roughly 225 times more efficient than F plasmids without the anti-CRISPR. Anti-CRISPRs expressed from the T-strand’s lagging region or the leading region of the complementary strand led to considerably less efficient conjugation compared to expression from the T-strand’s leading region (Fig. 3b,c).

These findings indicate that the localization of anti-defence genes in the leading region is crucial for effectively counteracting recipient defence systems. We postulate that this stems from the need to express anti-defence genes very early during the transfer for efficient conjugation.

## Discussion

**Fig. 4:**
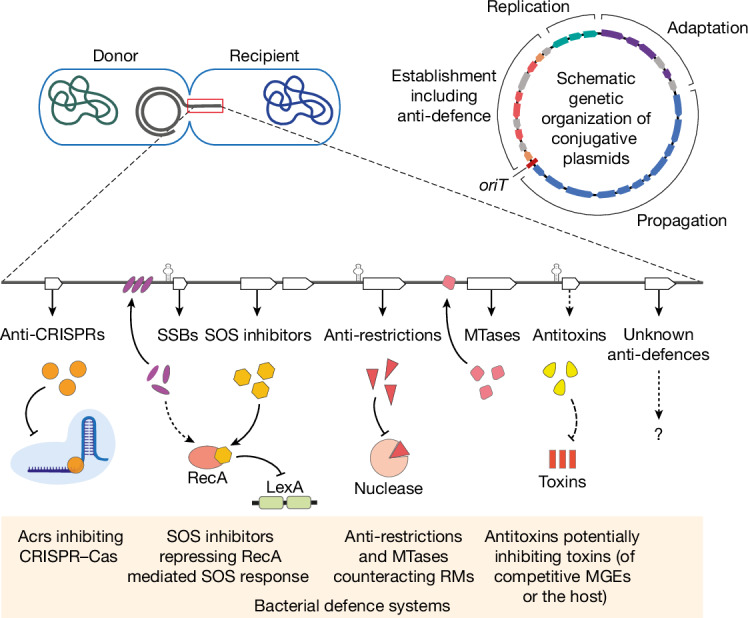
Proposed model of the plasmid protection by diverse defence evading systems encoded in the leading region. The figure illustrates how anti-defence genes with ssDNA promoters in the leading region can protect plasmids during the very early stages of transfer to a recipient cell. As the bacterial immune response activates defence systems against foreign DNA, various plasmid-encoded genes counteract these defences: anti-CRISPRs can inhibit CRISPR–Cas systems; SOS inhibitors (such as PsiB) can repress the cell’s SOS response by preventing RecA protein activation, thus inhibiting the cleavage of LexA, an SOS-response transcriptional repressor. Single-stranded binding (SSB) proteins, involved in the SOS-response inhibition, may protect transferred ssDNA from host nucleases. Anti-restriction proteins may prevent DNA cleavage by restriction–modification systems. MTases, methylating the transferred DNA can impede recognition by the host restriction–modification (RM) systems; and toxin–antitoxin genes potentially act against competitive MGEs or host defence systems (Supplementary Discussion). The top-right panel shows a schematic genetic organization of a conjugative plasmid. The four main functional gene groups are colour-coded: propagation (blue), adaptation (purple), replication (green) and the anti-defence genes (red) within the establishment module (orange).

An intrinsic part of the arms race between bacteria and MGEs is the interplay between defence and defence evasion systems. We present a broad and diverse set of plasmid-encoded anti-defence genes, reflecting the vast and dynamic repertoire of bacterial immune systems^3^. Examination of conjugative elements across extensive genomic and metagenomic datasets revealed a high concentration of anti-defence genes in the leading region. Our experiments confirmed the critical role of this region in overcoming host defences and enhancing conjugation efficiency. Although the genetic region adjacent to the propagation module (that is, mobility genes) and the *oriT* is at present termed the ‘establishment’ region^20,27,56^, our findings highlight that inhibiting host defences is a key function of genes in this region. We thus propose designating this region as ‘establishment and anti-defence’ (Fig. 4).

Plasmids have been explored as conjugative delivery systems for editing natural microbial communities^57^ and for various biotechnological applications, such as targeting antibiotic-resistant bacteria using CRISPR nucleases. However, these attempts often resulted in low conjugation efficiency, particularly in complex microbial communities such as the human gut^58–60^. These studies emphasize that improving conjugation efficiency is vital for future applications. Our findings may provide a crucial factor in understanding the set of genetic tools required for efficient conjugation-based delivery systems for medical and biotechnological applications.

## Methods

### Datasets and initial annotation

The assemblies of all genomes and metagenomes from NCBI whole-genome projects^61^ and all assembled metagenomes available from EBI MGnify were downloaded on 14 March 2020 (ref. ^62^). After excluding genomes from Metazoa, Fungi and Viridiplantae, the dataset included 596,338 genomes and 22,923 metagenomes from various ecosystems. This dataset contained more than 45 million contigs of at least 10 kilobase pairs. In WGS, 31,119 sequences were explicitly annotated as plasmids. Gene calling and initial annotation were performed using prodigal^63^ v.3.0.0 and Prokka^64^ v.1.14.6. As part of the annotation process, genes were assigned Kyoto Encyclopedia of Genes and Genomes (KEGG) orthologue groups as described in ref. ^65^. Briefly, all KEGG genes associated with a KEGG orthologue in the KEGG database downloaded on 14 May 2021 were subclustered with MMseqs2 (ref. ^66^). The pHMM database included all subclusters with more than five members after aligning the orthologues with MAFFT^67^ and building the model using HMMer suite’s hmmbuild (v.3.3.2)^68^. MOB classification to types (F, P1, T, V, C, Q, P2, H, B, P3 or M) was performed using pHMMs acquired from MOBscan^69^. Of these MOB types, P3 and M were not represented in our data.

### Relaxase/*traM* and *oriT* detection

Detection of relaxase and *traM* relaxosome genes was performed using hmmsearch^68^ (*e* value threshold 10^−6^) against all the proteins in our dataset. The pHMMs were acquired from Pfam^70^ and MOBscan^69^ databases (Supplementary Table 3). Contigs with more than two relaxase or TraM hits were filtered out. Known *oriT* sequences were retrieved from oriTfinder^71^ (1,075 *oriT* sequences), OriT-strast^72^ (112 sequences) and from ref. ^12^ (40 sequences). The search was conducted as in ref. ^12^, with the following differences: our approach incorporated, in addition to the experimentally validated *oriTs*, also computationally predicted *oriT* sequences from oriTfinder and BLAST’s word size was reduced to five for increased sensitivity. Specifically, we used BLAST+ (v.2.10.0)^73^, with an *e* value threshold of 10^−6^ and the following parameters ‘-task blastn-short -word_size 5’ against relaxase/*traM*-containing contigs (11,908 WGS plasmids and 1,019,093 genomic and metagenomic contigs). Known *oriT* sequences were detected in 5,304 annotated plasmids with relaxase and 238,363 relaxase-containing genomic/metagenomic contigs. In contigs with more than one *oriT* hit, the best-scoring *oriT* was considered. The distance between the relaxase/*traM* gene and the *oriT* was calculated as the number of nucleotides between the end of the relaxase/*traM* gene and the start of the *oriT*. Contigs in which this distance between the two was more than 3,500 bp were filtered out. We included contigs in which the *oriT* partially overlapped the relaxase gene, but cases in which the *oriT* was entirely contained within the relaxase gene were excluded. Contigs in which relaxase genes or the *oriT* were at the first or last annotated sequences were excluded as well. Both these cases were omitted because they impeded our ability to determine the relative location of the *oriT* and the relaxase/*traM* gene. Overall, this filtering process yielded 4,441 WGS annotated plasmids and 206,158 potential conjugative elements containing a relaxase/*traM* gene and an *oriT*.

### Deduplication of redundant sequences

To avoid artefacts resulting from redundant sequences, we clustered all 677,638 ORFs of the 4,441 WGS plasmid contigs containing relaxase and *oriT* using CD-HIT^74^ (v.4.6). The percentage of shared ORFs (according to the clustering) for each pair of contigs was calculated. If two plasmids shared more than 90% of the ORFs, the plasmid with fewer ORFs was filtered out. This process yielded 2,259 representative plasmids. This deduplication process was also applied to 206,158 potential conjugative elements identified in genomic and metagenomic sequences, yielding 26,327 non-redundant contigs of potential conjugative elements. Combining the annotated plasmids with the rest of the potential conjugative elements and removing plasmids appearing in both sets resulted in a total of 27,677 non-redundant contigs of potential conjugative elements. The host phylogenetic distribution of these non-redundant contigs (Extended Data Fig. 3) was mapped to the bacterial subtree from iTOL^75^, which is based on a concatenated alignment of 31 protein families related to translation and transcription^76^. The tree visualization was generated using ggtreeExtra (v.1.8.1)^77^.

### Anti-defence and mobility gene annotation

Protein families with known anti-defence functions were modelled using 139 pHMMs (based on sequences detailed in Supplementary Table 3). To characterize the plasmid’s transfer genes, we searched for conjugation proteins using pHMMs downloaded from Pfam^70^ or computed on the basis of proteins from relevant KEGG orthologues^78^ (Supplementary Table 3). To annotate transposases, we used 49 pHMMs from TnpPred data archive^79^. Hmmsearch with an *e* value threshold of 10^−6^ was performed against the non-redundant set of potential conjugative elements containing a relaxase/*traM* and an *oriT*.

### Statistical enrichment analysis and ORF clustering

To test which ORF positions in the leading region of plasmids and potential conjugation elements were significantly enriched with anti-defence genes, we performed a Fisher’s exact test (one-sided, *P* < 0.001) on the anti-defence gene count at each location (anti-defence gene count versus the total number of genes). The enrichment analysis was performed on the 2,259 sequences of well-annotated plasmids for 965 positions with at least 50 ORFs and on each MOB type of the 26,327 sequences of potential conjugative elements. MOB types with at least 50 ORFs in the first ten positions were filtered out if they did not show a significant enrichment in most of these positions. This resulted in the omission of three MOB types: T, C and P2, comprising 5,686 sequences. After removing these MOB types, we continued the analysis focusing on MOB types F, P1, V, Q, H and B, which were identified in 21,907 non-redundant plasmids and potential conjugative elements. For the 5,958 positions in this set that had at least 50 ORFs, we performed the anti-defence enrichment test. The same test was also conducted separately for each anti-defence category (namely anti-CRISPRs, anti-restriction genes and SOS inhibitors). The *P* values of all statistical analyses were corrected for multiple testing using the false discovery rate (FDR) (*α* = 0.001).

The leading region genes (ORFs in positions 1–28) of the 21,907 non-redundant potential conjugative sequences were clustered using MMseqs2 (ref. ^66^) (with sensitivity 7.5 and coverage 0.5). We examined the 300 largest gene families, which had more than 170 ORFs each (combined, they contained a total of 205,296 ORFs). Each family was aligned using MAFFT^67^ (v.7.475), and a pHMM was constructed from each alignment (Supplementary Data 1). Hmmsearch (*e* value threshold 10^−6^) was performed using these pHMMs against all potential conjugative sequences. To statistically test the enrichment of each gene family in the 1–28 ORF positions of the leading region, we performed a one-sided Fisher’s exact test and FDR correction. Forty-five of the 300 gene families were not significantly enriched in the leading regions (*α* = 0.001) and thus omitted from downstream analyses. The *P* value for each gene family after correction for multiple testing is specified in Supplementary Table 1.

The 188,655 proteins associated with families with at least five members were annotated on the basis of their DIAMOND^80^ hits (with *e* value < 10^−6^, coverage 0.6) against UniprotKB^81^. We examined the orientation of the ORFs relative to the *oriT* position in each of the significantly enriched gene families. The overall orientation of a gene family was defined on the basis of most of its ORFs. In families with ORFs that received different annotations, the most frequent annotation was used (Supplementary Table 1). For the 100 most prevalent families that were statistically enriched in the leading region, we also searched for known conserved domains using NCBI CDD^82^ (*e* < 10^−6^), NCBI-nr^83^ (*e* < 10^−6^) and HHpred^84^ (against the PDB^85^ and Pfam^70^ databases and *e* value threshold of 10^−10^).

### Structural analysis

Structural prediction for 107,893 uncharacterized proteins smaller than 900 amino acids from gene families with at least five members was carried out using ESMfold^86^. The structure of 128 known anti-defence genes (115 anti-CRISPR and 13 anti-restriction genes) was conducted in the same manner. Subsequently, we used Foldseek^87^ to search the predicted structures against the UniProt50 Foldseek structural database, encompassing 53.7 million non-redundant proteins^87^, as well as against the database of the 128 anti-defence protein structures we predicted. Visualization of protein structures was performed with UCSF ChimeraX^88^.

### F*rpo* and *ssi* promoter identification

To identify known F*rpo*/*ssi* sequences in the anti-defence islands, we created a BLAST^73^ dataset of all the gene regulatory regions with lengths of 50–350 bp in the leading regions of potential conjugative elements. We performed a BLAST search (BLAST+ v.2.10.0, *e* value threshold 10^−6^) against the five known F*rpo*/*ssi* sequences^18,89^ (Supplementary Table 2).

New candidate F*rpo* sequences were detected by seeking the consensus sequences of the −35, −10 (5′-TTGACA-3′ and 5′-TATAAT-3′, respectively) and the A + T rich UP-element located upstream of the −35 element^90^, in the intergenic regions of the islands represented in Fig. 2a–d. We then performed a BLAST search of the putative F*rpo* candidates from these islands against all the leading regions of our set of potential conjugative elements.

The DNA secondary structures of the F*rpo*/*ssi* elements were predicted using the RNAfold web server with the 2004 David H. Mathews model for DNA^91,92^. The graphical illustrations of the DNA structures (Fig. 2e,f) were produced using RNAtist^93^.

### Bacterial strains, plasmids and growth

Bacterial strains, plasmids, gRNA sequences and oligonucleotides are detailed in Supplementary Table 4. The *E. coli* strains were routinely cultured in Luria–Bertani (LB) medium at 30 or 37 °C supplemented with antibiotics at the following concentrations: tetracycline (10 µg ml^−1^), streptomycin (100 µg ml^−1^), chloramphenicol (25 µg ml^−1^), kanamycin (50 µg ml^−1^) and carbenicillin (100 µg ml^−1^).

Gene cloning into the F plasmid was performed using lambda Red recombination^94^. Modified F plasmids were transferred to the background strain K12 MG1655 *rpsL* (StrepR) by means of conjugation (detailed below). The SpCas9 sequence was amplified from Addgene plasmid no. 101044, followed by cloning into the pD5 vector using Gibson Assembly. The insertion of gRNAs was performed by PCR amplification using primers that included the gRNA sequences and ligation of the products, followed by electroporation into DH10β and K12 MG1655 *E. coli* using a room temperature protocol^95^ and verified by Sanger sequencing.

### Conjugation assays

Overnight cultures of recipient and donor cells grown on LB and selective antibiotics (tetracycline for the donors and kanamycin for recipients) were diluted 1:100 and grown to an optical density at 600 nm of 0.4. The cells were washed once with LB (2 min, 9,000 rpm) and resuspended with 50 µl of LB per conjugation. Donor (30 µl) and recipient (30 µl) cultures were mixed, and 20 µl of the mix was plated on an LB agar plate with 0.05 mM arabinose for the activation of SpCas9, then incubated for 2 h at 37 °C. Following incubation, cells were resuspended from the agar with 600 µl of 1× PBS, serially diluted 1:5 and plated on LB agar supplemented with 0.05 mM arabinose and the appropriate antibiotics to select for the recipient (R) or transconjugant (T) populations. The transconjugant frequency was quantified as T/(R + T). The conjugation efficiency was determined by calculating the transconjugant frequency per conjugation divided by the transconjugant frequency of the control, that is, the conjugation of the F plasmid without *acrIIA4* into recipients expressing non-targeting SpCas9. The plate with transconjugant colonies (Fig. 3b) was photographed using PhenoBooth+ (Singer Instruments).

### Reporting summary

Further information on research design is available in the Nature Portfolio Reporting Summary linked to this article.

## Online content

Any methods, additional references, Nature Portfolio reporting summaries, source data, extended data, supplementary information, acknowledgements, peer review information; details of author contributions and competing interests; and statements of data and code availability are available at 10.1038/s41586-024-07994-w.

## Supplementary information

Supplementary Discussion Discussion on the potential roles of toxin–antitoxin genes in the leading region of plasmids and their establishment.

Reporting Summary

Supplementary Table 1 Statistical analysis for 300 largest protein families, alongside sequence-based annotation and structural analysis of 100 largest protein families, and unannotated families enriched in the leading region.

Supplementary Table 2 F*rpo*-like sequences used in this study.

Supplementary Table 3 Anti-defence and mobility genes used in this study.

Supplementary Table 4 Strains, plasmids and oligonucleotides used in this study.

Supplementary Table 5 List of 27,677 non-redundant plasmids and potential conjugative elements retrieved from genomic and metagenomic databases, and 21,907 non-redundant elements after excluding MOB types T, P2 and C.

Supplementary Table 6 ORF accessions of genes associated with families of at least five members enriched in the leading region.

Supplementary Data 1 pHMMs for the largest 300 gene families enriched in the leading region.

Supplementary Data 2 Protein sequences associated with families enriched in the leading region.

Supplementary Data 3 Sequence of the F*rpo*-*acrIIA4*-*cat* construct used in this study.

## Source data

Source Data Fig. 1

Source Data Fig. 3

Source Data Extended Data Fig. 2

## Figures and Tables

**Extended Data Table 1:**
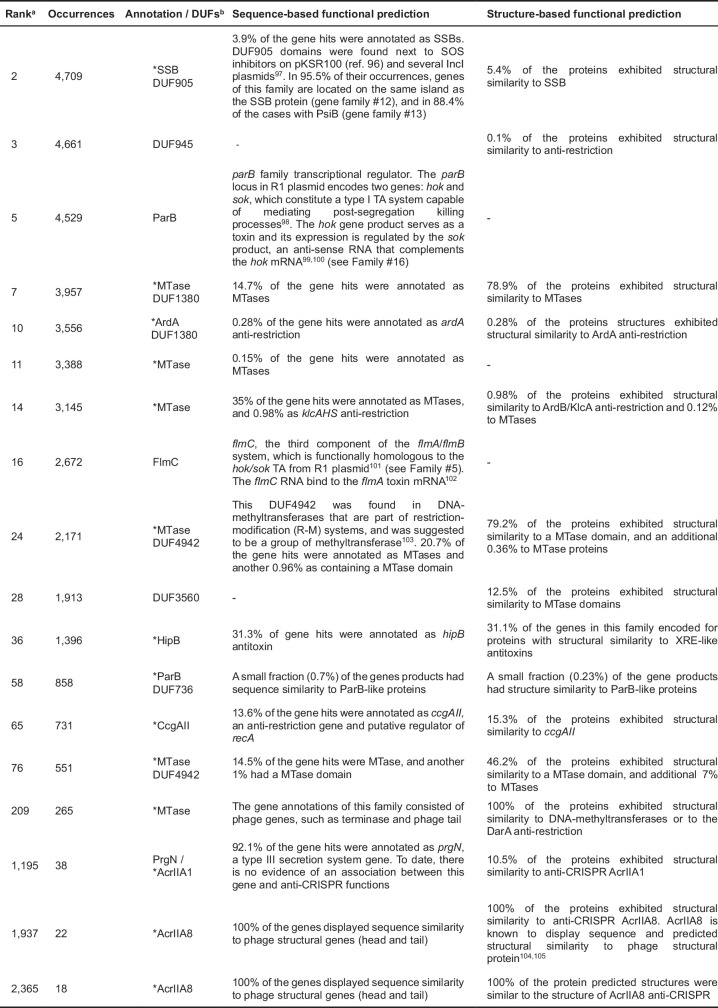
Prevalent uncharacterized gene families in the leading region with putative anti-defence functions

^a^ Based on the gene family size. ^b^ Presence of domains of unknown functions. Refs. ^96–105^. * Asterisks note putative annotations.

## Data Availability

All the analyses are based on publicly available, previously published datasets. The accessions of the analysed sequences are listed in Supplementary Table 5. The gene family numbers and ORF accessions are documented in Supplementary Table 6. The protein sequences of ORFs associated with gene family enriched in the leading region are provided in Supplementary Data 2. Profile HMMs produced as part of this study are available in Supplementary Data 1. Source data are provided with this paper.

## Code availability

This paper does not report the development of original code.
