# Supplementary material for: Diverse anti-defence systems are encoded in the leading region of plasmids
Source: Nature. 2024 Oct 9;635(8037):186–92. doi: 10.1038/s41586-024-07994-w (PMC11541004; doi:10.1038/s41586-024-07994-w)
Supplement: Supplementary file 1 — Discussion on the potential roles of toxin–antitoxin genes in the leading region of plasmids and their establishment. [file 41586_2024_7994_MOESM1_ESM.pdf]

nature portfolio

https://doi.org/10.1038/s41586-024-07994-w

Supplementary information

Diverse anti-defence systems are encoded in the leading region of plasmids

In the format provided by the authors and unedited

Supplementary Discussion

The overrepresentation of toxin-antitoxin (TA) genes in the leading region suggests a role in plasmid establishment. We propose three potential mechanisms: (i) TA systems, known to function as "addiction systems" via post-segregation killing (PSK)[106,107], may induce cell death if a conjugative element fails to establish. This would indirectly protect plasmids from degradation by host defence systems. (ii) TA systems, demonstrated to act as defence systems against other MGEs[108,109], could proactively facilitate plasmid establishment in the presence of competitive MGEs[110,111]. Plasmids encoding TA systems have shown a competitive advantage over those lacking them[109,112], and can also inhibit phages, which have been suggested to impede conjugation[108,113,114]. For instance, the ToxN/ToxI TA system from plasmid pECA1039 inhibits phage φA2 and φM1[115], while the AbiEii/AbiEi system from plasmid pNP40 inhibits the 936 phage family[116]. (iii) Antitoxins, as reported in phages, may counteract TA systems functioning as defence mechanisms[117,118]. For example, the Dmd protein of bacteriophage T4 acts as an antitoxin against *Escherichia coli* LsoA and RnlA toxins[118]. Additionally, the ADP-ribosyltransferase Alt of T4 bacteriophage can chemically alter and decrease the cleavage activity of the *Escherichia coli* MazF toxin[117]. This MazF modification occurs immediately following T4 infection, suggesting early expression during phage infection[117]. This hypothesis assumes TA systems can function as anti-plasmid defence systems, however, it should be noted that no such function has been reported to date.

Supplementary References

106. Gerdes, K., Rasmussen, P. B. & Molin, S. Unique type of plasmid maintenance function: postsegregational killing of plasmid-free cells. Proceedings of the National Academy of Sciences 83, 3116–3120 (1986).

107. Hernández-Arriaga, A. M., Chan, W. T., Espinosa, M. & Díaz-Orejas, R. Conditional Activation of Toxin-Antitoxin Systems: Postsegregational Killing and Beyond. *Microbiol. Spectr.* **2**, 10.1128/microbiolspec.plas-0009–2013 (2014).

108. Song, S. & Wood, T. K. A Primary Physiological Role of Toxin/Antitoxin Systems Is Phage Inhibition. *Front. Microbiol.* **11**, (2020).

109. Cooper, T. F., Paixão, T. & Heinemann, J. A. Within-host competition selects for plasmid-encoded toxin–antitoxin systems. *Proc. R. Soc. B Biol. Sci.* **277**, 3149–3155 (2010).

110. Pinilla-Redondo, R. *et al.* Type IV CRISPR–Cas systems are highly diverse and involved in competition between plasmids. *Nucleic Acids Res.* **48**, 2000–2012 (2020).

111. Igler, C., Huisman, J. S., Siedentop, B., Bonhoeffer, S. & Lehtinen, S. Plasmid co-infection: linking biological mechanisms to ecological and evolutionary dynamics. *Philos. Trans. R. Soc. B Biol. Sci.* **377**, (2022).

112. Cooper, T. F. & Heinemann, J. A. Postsegregational killing does not increase plasmid stability but acts to mediate the exclusion of competing plasmids. *Proc. Natl. Acad. Sci.* **97**, 12643–12648 (2000).

113. Harrison, E. et al. Bacteriophages Limit the Existence Conditions for Conjugative Plasmids. mBio 6, 10.1128/mbio.00586-15 (2015).

114. Igler, C., Schwyter, L., Gehrig, D. & Wendling, C. C. Conjugative plasmid transfer is limited by prophages but can be overcome by high conjugation rates. *Philos. Trans. R. Soc. B Biol. Sci.* **377**, 20200470 (2022).

115. Fineran, P. C. *et al.* The phage abortive infection system, ToxIN, functions as a protein–RNA toxin–antitoxin pair. *Proc. Natl. Acad. Sci.* **106**, 894–899 (2009).

116. Dy, R. L., Przybilski, R., Semeijn, K., Salmond, G. P. C. & Fineran, P. C. A widespread bacteriophage abortive infection system functions through a Type IV toxin–antitoxin mechanism. *Nucleic Acids Res.* **42**, 4590–4605 (2014).

117. Alawneh, A. M., Qi, D., Yonesaki, T. & Otsuka, Y. An ADP-ribosyltransferase Alt of bacteriophage T4 negatively regulates the *Escherichia coli* MazF toxin of a toxin–antitoxin module. *Mol. Microbiol.* **99**, 188–198 (2016).

118. Otsuka, Y. & Yonesaki, T. Dmd of bacteriophage T4 functions as an antitoxin against *Escherichia coli* LsoA and RnlA toxins. *Mol. Microbiol.* **83**, 669–681 (2012).
