# Supplementary material for: Diverse anti-defence systems are encoded in the leading region of plasmids
Source: Nature. 2024 Oct 9;635(8037):186–92. doi: 10.1038/s41586-024-07994-w (PMC11541004; doi:10.1038/s41586-024-07994-w)
Supplement: Supplementary file 2 — Reporting Summary [file 41586_2024_7994_MOESM2_ESM.pdf]

nature portfolio

Corresponding author(s): Dr. David Burstein

Last updated by author(s): Aug 8, 2024

# Reporting Summary

Nature Portfolio wishes to improve the reproducibility of the work that we publish. This form provides structure for consistency and transparency in reporting. For further information on Nature Portfolio policies, see our Editorial Policies and the Editorial Policy Checklist.

## Statistics

For all statistical analyses, confirm that the following items are present in the figure legend, table legend, main text, or Methods section.

| n/a | Confirmed | |
|---|---|---|
| ☐ | ☒ | The exact sample size (*n*) for each experimental group/condition, given as a discrete number and unit of measurement |
| ☐ | ☒ | A statement on whether measurements were taken from distinct samples or whether the same sample was measured repeatedly |
| ☐ | ☒ | The statistical test(s) used AND whether they are one- or two-sided<br>*Only common tests should be described solely by name; describe more complex techniques in the Methods section.* |
| ☒ | ☐ | A description of all covariates tested |
| ☒ | ☐ | A description of any assumptions or corrections, such as tests of normality and adjustment for multiple comparisons |
| ☐ | ☒ | A full description of the statistical parameters including central tendency (e.g. means) or other basic estimates (e.g. regression coefficient) AND variation (e.g. standard deviation) or associated estimates of uncertainty (e.g. confidence intervals) |
| ☐ | ☒ | For null hypothesis testing, the test statistic (e.g. *F*, *t*, *r*) with confidence intervals, effect sizes, degrees of freedom and *P* value noted<br>*Give P values as exact values whenever suitable.* |
| ☒ | ☐ | For Bayesian analysis, information on the choice of priors and Markov chain Monte Carlo settings |
| ☒ | ☐ | For hierarchical and complex designs, identification of the appropriate level for tests and full reporting of outcomes |
| ☒ | ☐ | Estimates of effect sizes (e.g. Cohen's *d*, Pearson's *r*), indicating how they were calculated |

*Our web collection on statistics for biologists contains articles on many of the points above.*

## Software and code

Policy information about availability of computer code

| | |
|---|---|
| Data collection | The following tools were used for data collection (oriT sequences): oriTfinder (v.1.1) and OriT-strast (v.3.0) |
| Data analysis | The following tools were used for the data analysis: BLAST (BLAST+ 2.10.0), HMMer (v.3.3.2), MAFFT (v.7.475), prodigal (v.3.0.0), Prokka (v.1.14.6.), CD-HIT (v.4.6), NCBI-CDD (v.3.20), HHpred (v.57c8707149031cc9f8edceba362c71a3762bdbf), MMseqs2 (v.bad16c765aac60d84a8fde3548adbb06b34980bd), RNAfold web server (v.2.5.1), RNAtistCore (https://github.com/fjossinet/RNArtistCore, v.0.3.3), DIAMOND (v.2.0.11.149), ESMfold (v.2.0.1), Foldseek (v.7d0c07f89a8f254789342dc041726cfc1c7223e1), UCSF ChimeraX (v.1.7.1; 2024-01-23), ggtreeExtra (v.1.8.1). |

For manuscripts utilizing custom algorithms or software that are central to the research but not yet described in published literature, software must be made available to editors and reviewers. We strongly encourage code deposition in a community repository (e.g. GitHub). See the Nature Portfolio guidelines for submitting code & software for further information.

## Data

Policy information about availability of data

All manuscripts must include a data availability statement. This statement should provide the following information, where applicable:

- Accession codes, unique identifiers, or web links for publicly available datasets
- A description of any restrictions on data availability
- For clinical datasets or third party data, please ensure that the statement adheres to our policy

All the analyses are based on publicly available, previously published datasets. The assemblies of all genomes and metagenomes from NCBI whole-genome projects (WGS) and all assembled metagenomes available from EBI Mgnify were downloaded on March 14, 2020. KEGG genes associated with a KO in the KEGG database downloaded on May 14, 2021. Structures from UniProt50 Foldseek database were downloaded on May 16, 2023.
Profile HMMs associated with the manuscript were downloaded from KEGG orthologs on March 14, 2020, Pfam database on June 11, 2021; TnpPred data archive (https://www.mobilomics.org/software/tnppred/downloads.php) on June 20, 2021; and MOBscan data archive on May 5, 2021.
Profile HMMs produced as part of this study are available in Supplementary Data 1. The accessions of the analyzed sequences are listed in Supplementary Table 5. The gene family numbers and ORF accessions are documented in Supplementary Table 6. The protein sequences of ORFs associated with gene family enriched in the leading region are provided in Supplementary Data 2.

## Research involving human participants, their data, or biological material

Policy information about studies with human participants or human data. See also policy information about sex, gender (identity/presentation), and sexual orientation and race, ethnicity and racism.

| | |
|---|---|
| Reporting on sex and gender | N/A |
| Reporting on race, ethnicity, or other socially relevant groupings | N/A |
| Population characteristics | N/A |
| Recruitment | N/A |
| Ethics oversight | N/A |

Note that full information on the approval of the study protocol must also be provided in the manuscript.

# Field-specific reporting

Please select the one below that is the best fit for your research. If you are not sure, read the appropriate sections before making your selection.

☒ Life sciences ☐ Behavioural & social sciences ☐ Ecological, evolutionary & environmental sciences

For a reference copy of the document with all sections, see nature.com/documents/nr-reporting-summary-flat.pdf

# Life sciences study design

All studies must disclose on these points even when the disclosure is negative.

| | |
|---|---|
| Sample size | N/A: No sampling was performed in this study |
| Data exclusions | N/A: No data points were excluded |
| Replication | In the conjugation experiments, standard errors were calculated from three biologically independent experiments. |
| Randomization | N/A: Experiments used predefined bacterial strains and plasmids. |
| Blinding | Not applicable<br>N/A: Study outcomes were based on quantitative measurements of bacterial growth and plasmid transfer |

# Reporting for specific materials, systems and methods

We require information from authors about some types of materials, experimental systems and methods used in many studies. Here, indicate whether each material, system or method listed is relevant to your study. If you are not sure if a list item applies to your research, read the appropriate section before selecting a response.

## Materials & experimental systems

| n/a | Involved in the study |
|---|---|
| ☒ | ☐ Antibodies |
| ☒ | ☐ Eukaryotic cell lines |
| ☒ | ☐ Palaeontology and archaeology |
| ☒ | ☐ Animals and other organisms |
| ☒ | ☐ Clinical data |
| ☒ | ☐ Dual use research of concern |
| ☒ | ☐ Plants |

## Methods

| n/a | Involved in the study |
|---|---|
| ☒ | ☐ ChIP-seq |
| ☒ | ☐ Flow cytometry |
| ☒ | ☐ MRI-based neuroimaging |

# Plants

| | |
|---|---|
| Seed stocks | N/A |
| Novel plant genotypes | N/A |
| Authentication | N/A |
